# Supplementary material for: Modeling sickness absence data: A scoping review
Source: PLoS One. 2020 Sep 15;15(9):e0238981. doi: 10.1371/journal.pone.0238981 (PMC7491724; doi:10.1371/journal.pone.0238981)
Supplement: Attachment — Submitted filename: PRISMA-review.pdf [file pone.0238981.s003.pdf]

Modeling sickness absence data: a scoping review

General comments

To study the determinants of the Sick Leave, this essay used the method called Scoping Review from PRISMA to help identify major trends and gaps in the scientific literature that could guide researchers towards better modeling.

At first, a systematic search was performed in five databases from different fields with three queries items : 1) Model 2) Absence 3) Work. Those different fields include databases: MEDLINE, World of Science, Science Direct, PsychInfo and Econlit. They were meant to target the articles focused on the study of sick leave from work. To reduce the error, all the articles considered to be clearly inappropriate were eliminated in this step and all the possible outcomes that might affect SL were kept.

Secondly, extract resources from the selected model by the categories:

1. Metadata: publication year, journal title, authors;
2. Dataset characteristics: study population, country of the study, first year of the study;
3. Statistical methods: statistical model, definition of the outcome, criteria for the evaluation of the models, predictive ability of the model (AUC or C-Index), consideration of the multilevel nature of SL data.

Finally, based on the extracted information from those included article, a descriptive analysis is performed to explore the trends and frequencies of those statistical approaches.

The findings in this article are that physical issues, mental issues, as well as different occupations might influence the result of SL and the models involved in those studies are

- Univariate SL outcome modeling:
    1. Count data: Poisson, Negative binomial...
    2. Time-to-Event data: Cox model and its derivations...
    3. Categorical data: Logistic regression with different link functions...
    4. Continuous Indicators: Linear regression, Machine Learning...
- Bivariate SL outcome modeling.
- Structural Equation modeling.

In the very end, this article also stated its limitation and potential improvement in the future, in terms of such as data collection, investgation on alternative statistical approaches,etc.

Specific comments

Major comments

- On page 5, line 81 to 82, in the criterion description, only the individual level involved SL studies were to be included. And models on the aggregated data would be considered irrelevant. Could it be explained why aggregated data on SL study is irrelevant in the search. Aggregated data could be fitted by a model talking about behaviors among different population's sick leave.
- In Figure 1, what is the relationship between those numbers, some numbers seemed to be able to be added up to get another number but it wasn't the fact, such as 469+416 is not 877. Could there be more introduction on those numbers?

Minor comments

- If an article appears on a peer-reviewed journal, to what extent, this article will be considered to be inappropriate. (Page 5,line 90)
- As mentioned, the Sick Leave is an interdisciplinary research theme, so is there any chance that the same model with the same approach will be published in different fields thus causing some duplication of collecting the articles.
- The determinants found to be likely to influence SL might be correlated to one another, such as physical issues and job types.
